# Textile materials inspired by structural colour in nature

**DOI:** 10.1039/d0ra01326a

**Published:** 2020-06-25

**Authors:** Celina Jones, Franz J. Wortmann, Helen F. Gleeson, Stephen G. Yeates

**Affiliations:** Department of Materials, The University of Manchester Manchester M13 9PL UK celina.jones@manchester.ac.uk franz.wortmann@manchester.ac.uk; School of Physics and Astronomy, University of Leeds Woodhouse Lane Leeds LS2 9JT UK h.f.gleeson@leeds.ac.uk; Department of Chemistry, The University of Manchester Oxford Road M13 9PL UK stephen.yeates@manchester.ac.uk

## Abstract

The concept of mimicking structural colour in nature as an alternative to traditional textile coloration techniques would reduce dependency on dyes, pigments and vast quantities of water in the textile supply chain. Structural colours originate from the physical interaction of light with nanoscale structures. This is exhibited in the bodies and wings of certain species of butterfly, beetles and plants. The angular optical effects of the *Chrysina gloriosa* beetle result from the periodicity due to the cholesteric liquid crystal (CLC) structure adopted by the cells in their exoskeleton. The optical properties of CLCs makes promising applications for optical sensors and anti-counterfeit materials. Application using inkjet printing technology enables designs to be tuned to meet product requirements, and with a hydrophobic treatment challenges associated with a rough surface such as textiles are overcome. Here we report inkjet printing CLC solutions onto hydrophobic pre-treated textiles. CIE L*a*b* values demonstrate the resultant colourful films display a greater degree of colour compared to those on untreated textiles.

## Abstract 2

The concept of mimicking structural colour in nature as an alternative to traditional textile coloration techniques would reduce dependency on dyes, pigments and vast quantities of water in the textile supply chain.

## Graphical abstract

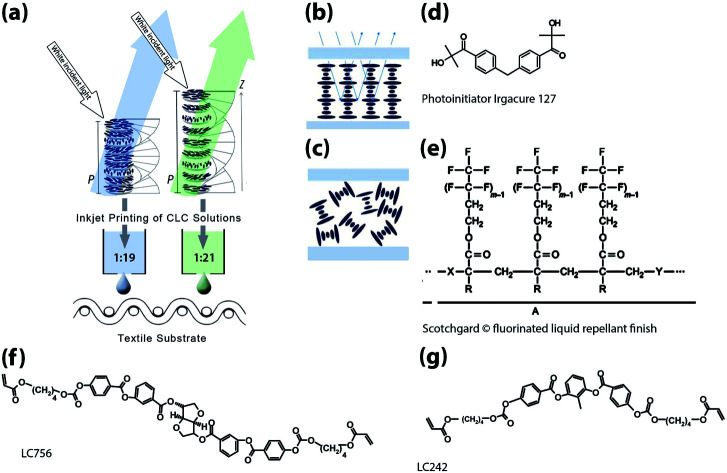

## 1 Introduction

Traditional textile colouration techniques require the observed material to absorb various wavelengths of visible light through the use of colorants, pigments and dyes. Colourant selection is dependent on fibre chemistry and method of application (batch dyeing, padding or printing for example), with successful execution dependent on pH, temperature and intensive water consumption.^1–3^

By contrast, structural colour works by the microscopic structure of the object scattering or reflecting various wavelengths of light resulting in the observer perceiving colour. However, with cholesteric liquid crystals (CLC, also known as chiral nematic LCs), half of incident unpolarized light is transmitted and the other half reflected. Typically, a black background substrate is used to absorb the transmitted light.

Of the reflected light, a specific band of wavelengths (central wavelength *λ*) is selected depending on the pitch (*p*) and average refractive index (*n*) such that *λ* = *np*. Used as a dopant, the chiral molecule can be added in small concentrations to alter the pitch of the mesogens in the mixture, thus producing a range of colours.

CLC polymers have been inkjet printed for stimuli-responsive materials such as optical sensors on smooth non porous substrates^4–10^ spray coated using a single solution for a reflective fibre^11^ and encapsulated in the production of flexible and drapable CLC textile displays.^12^ The optical effects produced by cholesteric liquid crystals has been exploited in the production of anticounterfeiting photonic inks.^13–15^ Ink-jet printing technology enables the possibility of creating a colourful textile print using a range of CLC solutions, and over-coming the design limitations of spray coating a singular solution.

**Fig. 1:**
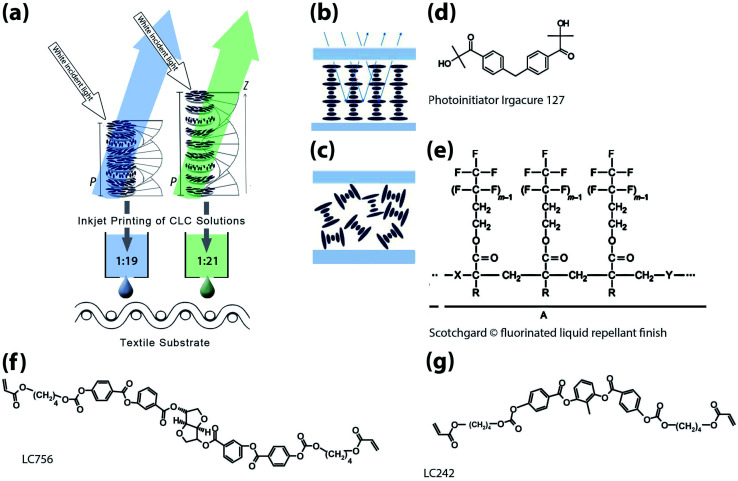
(a) Schematic showing inkjet printed cholesteric liquid crystal (CLC) solutions for structurally coloured textiles. Ratios of chiral dopant: nematic liquid crystal have been included in the schematic (b). Schematic showing selective wavelength reflection when there is planar alignment of CLC pitches as opposed to random alignment in (c).^16^ Molecular structures of (f) chiral dopant LC756 BASF (g) nematic liquid crystal LC242 and (d) Irgacure 127 photointiator combined with MEK solvent to create CLC solutions and (e) Scotchgard® fluorinated liquid repellant finish.^17^

As shown in schematic (a) in Fig. 1, this research explores for the first time the potential to ink-jet print CLC solutions producing coloured polymerised films onto rough porous substrates, such as textiles.

The orientation of the helix in these solutions, in relation to the substrate, is important. Controlling this is often known as alignment, defining the so-called texture. The optimum orientation is called planar alignment, also referred to as Grandjean, oily-streak, parallel and homogenous alignment^18^ (shown as Fig. 1(b)). Planar alignment enables the structure to optimally reflect the selected band of colour (wavelength). The application of a liquid repellent fluorochemical pre-treatment to the textile substrate could ensure the homogeneous alignment of the CLC pitches in the films by creating a smooth surface.

## 2 Experimental

### 2.1 Materials

White textured intermingled polyester 1/167/48 yarns were purchased from J. H. Ashworth and Son Ltd. Nematic liquid crystal LC242 BASF and chiral dopant LC756 BASF (shown in Fig. 1) are combined with UV initiator Irgacure 127® and MEK solvent (Sigma Aldrich). The chiral dopant concentration is adjusted to 5.05 wt% for blue (reflected wavelength of 457 nm) and 4.47 wt% for green (reflected wavelength of 523 nm) films. For the treated textile substrates a 3% 3 M Scotchgard® fluorinated liquid repellant finish was added using a pad mangle and then cured using W.Mathis AG CH-8155 oven at 170 °C for 60 seconds.

### 2.2 CLC film preparation

The polyester yarns are woven into a fabric with a plain weave structure containing 30 picks per centimeter. A 7.48 dense reed was used on a Northrop shuttle loom, L Model. A Hergeth Hollingsworth sample warper machine was used to prepare the warp threads for weaving. Each warp thread is then threaded through the heald wires in the heald frames of the loom. Prior to dyeing the fabric is heat set for 45 seconds at 180 °C in W.Mathis AG CH-8155 oven to stabilise the material and prevent shrinkage during the dyeing process. The white woven fabric is dyed black using 6% Dispersol Black XF. A liquor ratio of 15 : 1 was used (ml water to weight of fabric). A dye bath of pH 4–5 was used, adjusted with acetic acid. The substrate was dyed at high temperatures to promote the opening of the amorphous regions within the fibre structure. The temperature was raised to 130 °C at 2 °C per minute, and the textile substrate was dyed for 60 minutes. A Fuji Dimatix printer (DMP-2800) was used to print 200 picoliter drop volume of the CLC mixtures onto the fabric. The droplet spacing was set to 30 μm. The temperature of the substrate and printing head were set to 53 °C. The inkjet cartridge was set to 32 °C. The printing area was 10 mm × 10 mm. The angle of the cartridge head was 6.8° and jets 5–11 were selected for firing (7 jets in total). The jetting voltage used was 35–40 kHz with 1 V increments. A cleaning cycle was also implemented every 100 bands to ensure a homogeneous print was produced and to prevent clogging of the print head. After printing, photopolymerization was performed in a Fusion Systems Corporation I300MB (Heraeus Noblelight Fusion UV) UV curing machine for 30 seconds.

### 2.3 Characterization

The surface wettability of the treated and untreated textile substrates were assessed using a Sessile Drop Goniometer measuring their contact angle (CA) with a droplet of blue CLC solution and the changes in measurements with time.

CIELAB tristimulus values and reflectance measurements (including and excluding specular reflection) were performed using a Datacolor 650 reflectance spectrophotometer with D/9 viewing geometry, 10° CIE Standard Observer and with CIE D65 standard illuminant. Measurements were taken by placing the samples in the path of the incident beam with the extra ultra small (EUS) aperture on and UV filter off. This spectrophotometer is typically used to colour match fabric samples, and can thus be used to measure rough, uneven and smooth surfaces.

Scanning electron microscopy (SEM) was used to analyse the film thickness of treated and untreated printed polyester plain woven fabrics. The samples were prepared by embedding the substrates in epoxy araldite resin. One cured, the samples were grinded and polished with silicon carbide waterproof abrasive paper, mono diamond compound paste (3 and 1 micron) and OPS colloidal silica.

## 3 Results and discussion

### 3.1 Surface characterisation

**Fig. 2:**
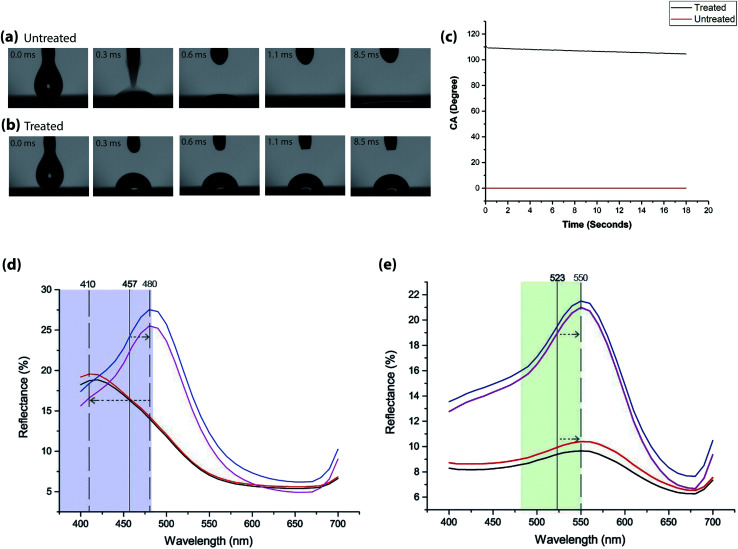
Impact of blue CLC solution on (a) untreated and (b) treated textiles. (c) Graph displaying surface wettability of the treated and untreated textile substrates by measuring contact angle (CA) with a droplet of blue CLC solution and the changes in measurements with time. (d) Reflectance values of 200 picoliters of blue CLC solution printed onto untreated and Scotchgard® treated plain woven polyester fabric. (e) Reflectance values of 200 picoliters of green CLC solution printed onto untreated and Scotchgard® treated plain woven polyester fabric.

The inkjet printing of blue CLC solution onto Scotchgard® treated plain woven polyester fabric shows a substantial increase in contact angle (CA) of up to 109.8° as shown in Fig. 2(b) and the black line in Fig. 2(c). By contrast, whilst measuring the contact angle of untreated plain woven polyester fabric, the blue CLC solution was absorbed immediately, shown in Fig. 2(a) and the red line in Fig. 2(c).

### 3.2 Reflectance values

The percentage reflectance data for 200 picolitres of blue CLC solution in Fig. 2(d) shows a significant difference in the reflectance values for treated and untreated textiles, along with the positioning of the peaks from the predicted wavelength of light (457 nm).

A vertical line has been added to the graph to indicate this region, along with the blue region of the visible spectrum. Reflectance values are taken with specular reflectance (SPIN) and without specular reflectance (SPEX). The maximum percentage reflectance for the polymerized film on untreated fabric is at 410 nm with SPIN (red line) and SPEX (black line) whereas for the same amount of solution on Scotchgard® treated fabric is at 480 nm with SPIN (blue line) and SPEX (purple line).

This demonstrates that the film on Scotchgard® treated textile reflects more light to the observer, but that the colour reflected differs from that on the untreated textile. The peak for the CLC film on the Scotchgard® treated textile is situated towards the green region of the visible spectrum.

**Fig. 3:**
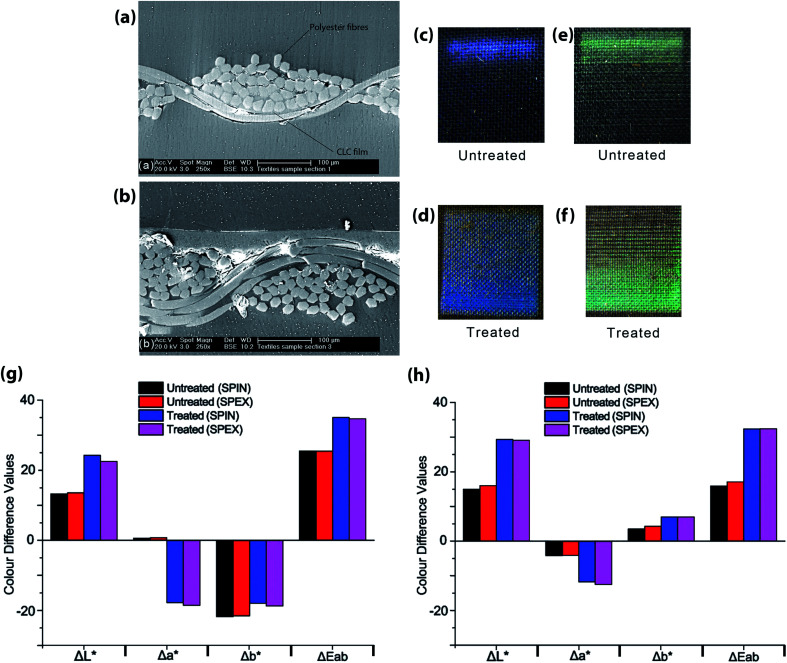
Images of textile cross section for (a) untreated and (b) Scotchgard® treated plain woven polyester with 200 picolitres of blue CLC solution taken on Philips XL30 FEG-SEM in backscattered electron mode. Photographs of untreated (c) and Scotchgard® treated (d) plain woven polyester with 200 picolitres of blue CLC solution. Photographs of untreated (e) and Scotchgard® treated (f) plain woven polyester with 200 picolitres of green CLC solution. (g) Graph comparing CIELAB values for differences in lightness (ΔL*), a* and b* values (Δa* and Δb*) and colour difference 
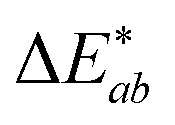
 of 200 picolitres of blue CLC solution from the black unprinted standards for treated and untreated plain woven polyester textiles. (h) Graph comparing CIELAB values for differences in lightness (ΔL*), a* and b* values (Δa* and Δb*) and colour difference 
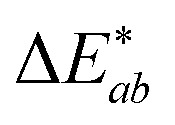
 of 200 picolitres of green CLC solution from the black unprinted standards for treated and untreated textiles.

The difference in values between specular included and excluded for the film created on the Scotchgard® treated textile, indicate a glossy or shiny film, which is not the case for the film printed on the untreated textile (as shown in Fig. 3(c) and (d)). The metallic appearance of some beetles occurs because of macroscopic, highly uniform areas of alignment of the helicoidal structure of chiral nematic LC polymers. Indeed some beetles can reflect 100% (not just 50%) of the light. More commonly, a chiral nematic LC polymer will have a domain structure, especially when coated onto a surface such as a textile. Fig. 3(b) shows that the length scale of the textile on which the LC polymer is deposited is of the order of a few microns. The non-uniformity of the helicoidal structure that is inevitable as it aligns on such surface features will have two effects. The boundaries between micron-sized domains will scatter light, giving a more matt appearance, and the slight differences in alignment will broaden the selective reflection peak. Both features contribute to the non-metallic appearance of the polymer LC deposited onto the textiles. The optical properties of a uniformly (or nearly uniform) film of polymer LC are well-known to be angularly dependent, and this feature may be of interest in some textile applications. The degree of angular dependence depends on the birefringence of the LC (controlled by the chemical structure) and the uniformity of the helicoidal structure. Both can be controlled, the former by material design and the second by surface treatment of the substrate (textile in this case) and the deposition method. Controlling details of the polarization and angular dependence of the pigment is a topic for future work. Other routes to synthetic structural colours have also been identified; whereas the chiral nematic polymers used here self-organise into a nanostructure that is identical to that found in certain beetles, it is also possible to produce small areas of diffractive structures reminiscent of those responsible for structural colour in some butterflies.^19^

The percentage reflectance data for 200 picolitres of green CLC solution in Fig. 2(e) shows a significant difference in the reflectance values for treated and untreated textiles, however the positioning of the peaks from the predicted wavelength of light (523 nm) are the same. A vertical line has been added to the graph to indicate this region, along with the green region of the visible spectrum. The maximum percentage reflectance for the polymerized film on untreated is 10% SPIN (red line) and SPEX (black line) at 550 nm whereas for the same amount of solution on Scotchgard® treated fabric is 21% at 550 nm SPEX (purple line) and SPIN (blue Line).

This demonstrates that the green film on treated textiles reflects more light to the observer, but that the colour reflected is the same. The peak for the CLC film on treated textiles is situated towards the yellow region of the visible spectrum. This is also supported by the prints shown in Fig. 3(e) for untreated and Fig. 3(f) treated.

The difference in values between specular included and excluded for the film created on Scotchgard® treated textiles indicate a glossy or shiny film, which is also the case for the film printed on untreated textiles.

When observing these films under an SEM microscope (shown in Fig. 3), the film has formed within the fabric structure for the untreated textile (a) visible in the photograph of the textile print (c). By contrast, the print has formed on the surface of the fabric structure for the treated textile shown for blue in (b) and (d) along with green prints in (e) and (f).

This would affect the thickness of the film produced, which according to the work carried out by Roberts *et al.*^20^ (developed from the works of St John *et al.*^21^) influences the reflectance spectra produced. The data in Fig. 2(d) supports these findings.

### 3.3 Colour measurement

The CIELAB (also referred to as L*a*b*) coordinates and colour space were developed in 1976 by the CIE to describe the lightness, hue and chroma of a sample. L* corresponds to the lightness of the sample, where the maximum value is 100 (relating to that produced by a reflecting diffuser) and the minimum is 0 (relating to black). The coordinates for a* and b* have positive and negative values, and each numerical value relates to a colour in the visible spectrum and is plotted in a three-dimensional colour space.

These coordinates can be used to calculate colour difference (Δ*E*) between two samples using the CIELAB formula in eqn (1).^22^1
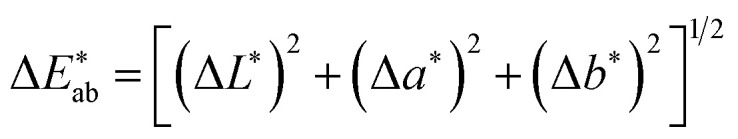


Lightness (ΔL*) differences can also be defined using eqn (2).2
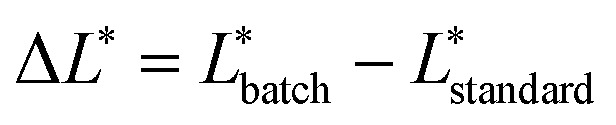


The values for 200 picolitres of blue CLC solution printed on untreated and treated polyester plain woven fabric are shown in Table 1.

**Table 1:** CIELAB values for CLC printed film produced from 200 picolitres of blue CLC solution on untreated and untreated polyester plain woven fabric

CLC solution	L*	a*	b*	ΔL*	Δa*	Δb*	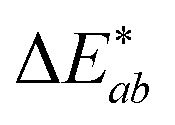
Untreated blue (SPIN)	34.2	0.6	−23.1	13.3	0.7	−21.8	25.5
Untreated blue (SPEX)	34.7	0.7	−23.1	13.6	0.8	−21.5	25.5
Treated blue (SPIN)	44.5	−17.9	−18.5	24.3	−17.8	−17.9	35.1
Treated blue (SPEX)	42.1	−18.7	−19.1	22.5	−18.6	−18.8	34.7

Fig. 3(g) shows that the CLC film printed onto treated textiles has a higher value for lightness difference (ΔL*) than that on untreated textiles. This may also be attributed to the increase in film thickness, and smoothness of the textile substrate from the CLC film.

The increase in magnitude of the negative Δa* values for treated textiles suggests that a greener film is created with the same amount of blue CLC solution than that printed on untreated textiles.

The Δb* values for untreated textiles suggest that a bluer film is created on this substrate, compared with that on Scotchgard® treated textiles.

The overall colour difference 
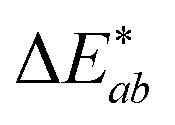
 for treated textiles is greater than that of untreated textiles from the unprinted black textile standard.

The values for 200 picolitres of green CLC solution printed on untreated and treated polyester plain woven fabric are shown in Table 2.

**Table 2:** CIELAB values for CLC printed film produced from 200 picolitres of green CLC solution on untreated and untreated polyester plain woven fabric

CLC solution	L*	a*	b*	ΔL*	Δa*	Δb*	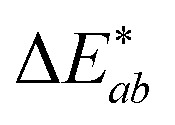
Untreated green (SPIN)	35.9	−4.2	2.2	14.9	−4.1	3.5	15.9
Untreated green (SPEX)	37.1	−4.1	2.8	16.0	−4.1	4.3	17.0
Treated green (SPIN)	49.5	−11.9	6.4	29.4	−11.8	6.9	32.3
Treated green (SPEX)	48.7	−12.6	6.6	29.1	−12.5	6.9	32.4

Fig. 3(h) compares the values for the difference in lightness (ΔL*), a* and b* values (Δa* and Δb*) and colour difference 
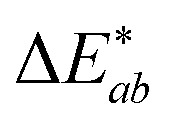
 of 200 picolitres of green CLC solution from the black unprinted standards for treated and untreated textiles.

Fig. 3(h) shows that the CLC film printed onto treated textiles has a higher value for lightness difference (ΔL*) than that on untreated textiles (including and excluding specular). This may also be attributed to the increase in film thickness, and smoothness of the textile substrate from the CLC film.

The increase in magnitude of the negative Δa* values for treated textiles suggests that a greener film is created with the same amount of green CLC solution than that printed on untreated textiles.

The Δb* values for Scotchgard® treated textiles suggest that a yellower film is created on this substrate, compared with that on untreated textiles.

The overall colour difference 
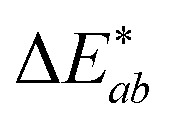
 for treated textiles is greater than that of untreated textiles, from the unprinted black textile standard.

## 4 Conclusions

Through biomimicry, structural colour exhibited in nature could be used to help solve design challenges in the textile industry. Interestingly the man-made structural colour here (a chiral nematic polymer liquid crystal) has exactly the same origin as the structural colour in some beetles. This offers further nuances in the effects obtained that could be investigated in future.

We report the potential to ink-jet print CLC polymerized films to rough porous substrates, such as textiles for the first time. The Scotchgard® treated textiles with inkjet printed CLC solutions improved the colour of the polymerised CLC films produced and ensured a homogenous alignment of the CLC pitches in the film. The inkjet printing processes enables a specific amount of CLC to be printed in a precise location, and the potential to digitally print a design containing a variety of colours using solutions created simply by varying the quantity of chiral dopant. In future work, with two cartridges (one containing the chiral dopant and the other with the nematic liquid crystal) it may be possible to use the ratios of these monomers to create a chromaticity diagram to achieve a range of desired colours. Steps to the removal of the black dyed substrate would progress this research from a reduction in dye and pigment dependency to the withdrawal of the dye kitchen from the textile supply chain. However, it is worth noting that the chiral materials discussed here will also give interesting structural colour effects on any background. Furthermore, in future work, the impact of these prints on textile properties such as drape, softness and performance such as colour fastness, should be explored.

## Conflicts of interest

There are no conflicts to declare.

## Supplementary Material
